# Difficulties in recruitment for a randomized controlled trial involving hysterosalpingography

**DOI:** 10.1186/1742-4755-3-5

**Published:** 2006-06-13

**Authors:** Denise AM Perquin, Anton JM de Craen, Frans M Helmerhorst

**Affiliations:** 1 Department of Obstetrics and Gynecology, Medical Centre Haaglanden, P.O. Box 432, 2501 CK, The Hague, The Netherlands; 2 Department of Gerontology and Geriatrics, Leiden University Medical Centre, P.O. Box 9600, 2300 RC Leiden, The Netherlands; 3 Department of Gynecology, Division of Reproductive Medicine, Leiden University Medical Centre, P.O. Box 9600, 2300 RC Leiden, The Netherlands

## Abstract

**Background:**

The usefulness of hysterosalpingography (HSG) as routine investigation in the fertility work-up prior to laparoscopy and dye had been assessed in a randomized controlled trial. Recruiting subjects to the study was more difficult than anticipated. The objective of this study was to explore possible reasons for non-participation in the trial.

**Methods:**

All newly referred subfertile women admitted to the Reproductive Medicine Clinic of Leiden University Medical Centre between 1 April 1997 and 31 December 1999, were eligible for the study. The reasons for non-participation were evaluated by scrutinizing the medical records.

**Results:**

Out of 759 women, a total of 127 (17%) agreed to participate in the trial. The most important reason for non-participation was because of exclusion criteria (73%). Other reasons were inattentive clinicians (3%) and patient-associated reasons (24%). Patient refusal and indecisiveness to enroll in the study were the most common patient-associated reasons. The most frequently stated reason for trial refusal was reluctance to undergo laparoscopy and dye mainly due to issues related to anesthesia and scheduling of procedure.

**Conclusion:**

Almost three-quarters of recruitment difficulties in this study were due to unavoidable reasons. To overcome the remaining avoidable reasons for non-participation, attention should be paid to appropriate instruction of the study protocol to the participating doctors and to provide adequate information, in layman's terms, to the patients. Reminding patients by notes or telephone calls for attending the clinic are helpful. It may be contingent upon tracing the reasons of clinicians and patients for non-participation to improve enrollment during a trial.

## Background

Between April 1997 and April 2002 we performed a pragmatic [1] multicentre randomized controlled trial comparing two different diagnostic strategies in the routine fertility work-up [2]. The hysterosalpingography (HSG) group underwent HSG first. If HSG showed normal uterine cavity, patent tubes and no tubal pathology, laparoscopy and dye followed after six months. In case of suspected tubal pathology, laparoscopy was performed within one or two months after HSG. The laparoscopy group did not receive HSG but underwent laparoscopy and dye directly after randomization.

The power of the trial was based on randomization of 750 subfertile women. Recruitment of patients into the trial was more difficult than expected. We estimated the highest recruitment rate from Leiden University Medical Centre (LUMC). However, about halfway through the trial, we had only recruited 177 women instead of an estimated 375. To understand this low recruitment rate, we initiated the current study to find strategies to avoid the major reasons for non-participation which could be implemented during the second half of the study or later in other studies in reproductive medicine. This study identified potential eligible participants visiting one of the three participating hospitals (Leiden University Medical Centre) during the first half of the recruitment period.

## Methods

All women in our study participated in a multicentre randomized controlled trial with or without the performance of HSG to assess the usefulness of hysterosalpingography as routine investigation in the fertility work-up prior to laparoscopy and dye. Recruitment strategy, description of subjects, and main results of the trial have been published elsewhere [2]. In short, the trial was performed in one university hospital (Leiden University Medical Centre, Leiden) and two non-university teaching hospitals (Medical Centre Haaglanden, The Hague and Groene Hart Hospital, Gouda). All newly referred subfertile women who visited one of the three hospitals between April 1997 and April 2002 were eligible for inclusion in the trial. Exclusion criteria were a) subfertility less than 1 year, b) woman older than 37 years at time of first visit, c) anovulation in spite of clomifene citrate or bromocriptin use, d) abnormal semen analysis according to WHO criteria [3], or e) testing of tubal patency performed in the past. Women were asked to participate in the trial by their treating gynecologist at the time that HSG would normally be planned and informed consent was obtained. If the women refused to participate in the trial, the reason for non-participation was recorded. A computer-based 1:1 ratio randomization procedure was used to allocate the women into two groups; the HSG group or the laparoscopy group. Informed consent was obtained from all women. The Institutional Review Boards of each of the three hospitals approved all stages of the trial.

Recruitment of subjects into the trial was lower than expected. We elucidated this current study to explore the determinants of non-participation during the first half of the recruitment period among all potentially suitable subjects of Leiden University Medical Centre to find strategies to avoid the major reasons for non-participation. We reviewed the medical records from all newly referred subfertile women who visited the Reproductive Medicine Clinic of Leiden University Medical Centre from 1 April 1997 to 31 December 1999. The medical record of each subfertile couple contained either a sticker indicating that the woman participated in the study or documented the reasons for non-participation.

## Results

From 1 April 1997 to 31 December 1999, 759 newly referred subfertile women visited the Reproductive Medicine clinic of Leiden University Medical Centre. A total of 127 women (17%) met the inclusion criteria and agreed to participate in the trial, the remaining 632 did not. The unavoidable reasons (467 women; 73%) and avoidable reasons (165 women; 27%) for non-participation are summarized in table 1. Almost three-quarters of the women did not participate due to exclusion criteria (73%), 3% due to inattentive doctors and the remaining 153 women (24%) due to patient-associated reasons. From these 153 women, 72 of them refused and 19 women were indecisive to enroll in the study. Fifty women never showed up for randomization after the initial visit. Personal circumstances such as leaving the area and relationship problems were also reported (n = 7).

**Table 1:** Unavoidable and avoidable reasons for non-participation

Unavoidable reasons	N = 467	%
Exclusion criteria	460	73
Androgenic factor	173	
Tubal testing performed in the past	114	
Pregnant before randomization	91	
Women older than 37 years at first visit	55	
Anovulation	27	
Patient's reason	7	1
Personal circumstances	7	
		
Avoidable reasons	N = 165	%

Doctor's reason	19	3
Eligible, but not approached	19	
Patient's reason	146	23
Refused to participate	72^1^	
No show-up after initial visit	50	
Indecisiveness	19	
Language barrier	5	

^1^see Table 2

Table 2 shows reasons for trial refusal among the 72 women. The most frequent stated reason was reluctance to laparoscopy and dye (35 women; 49%). Twenty-seven women (37%) did not state a reason for non-participation.

**Table 2:** Patient's reasons for trial refusal

Reasons	N = 72	%
Reluctance to laparoscopy and dye:	35	49
General anesthesia	20	
The timing of the laparoscopy is too soon	15	
Reluctance to hysterosalpingography:	3	4
Fear for pain	3	
Don't want to be involved in a research project	7	10
Reasons not documented	27	37

## Discussion

In retrospect, it seems clear that we had too optimistic recruitment targets. Main unavoidable reasons for non-participation in the trial were not meeting the inclusion criteria and personal circumstances. In 19 of 165 avoidable reasons for non-participation, it appeared that doctors were inattentive to approach their eligible patients for the trial. More details of their negligence were not documented, except that in general these doctors appeared to be willingly participating in the trial. Attention should be paid to appropriately instruct participating doctors in order to increase the recruitment of eligible patients. We have no evidence that physicians' preferences influenced the outcome of the randomized trial [4]. However, discussing the clinical relevance of the question as well as practical issues in the period that the protocol of the trial was designed appeared to be essential in the prevention of barriers in clinical recruitment [5].

Apprehensiveness towards one of the diagnostic procedures in this trial (laparoscopy) was mentioned by the women as the most prominent and avoidable reason for non-participation in the trial. General anesthesia prior to laparoscopy appeared to be a main obstacle for enrolment in the trial. Providing more adequate information on the actual procedure and using layman's terms may improve the rate of participation in such a trial. Although well educated and employed persons were more likely to refuse randomization because of preference [4], we think that providing more information focused on problems that may emerge from questionnaires disseminated among potential participants in the development of the trial, may optimize recruitment. Some patients did not wish to be involved in a research project. Once patients have made up their mind and once they have prepared a distinct preference, it is nearly impossible to persuade them for enrolment [6].

One shortcoming of our paper is that we studied the major reasons of non-participation of potentially eligible participants visiting only one hospital. Unavoidable reasons of non-recruitment accounted for three quarters of the non-participation. The exclusion factor might be higher in an academic centre due to specific criteria for referrals. The referred subfertile couples could have been older, with severe androgenic pathology or proven tubal pathology needing specialized assisted reproductive treatments. Another objective of this study was to find strategies to avoid the major reasons for non-participation which could be implemented during the second half of the study or later in other studies focusing on reproductive medicine. We assume that the major avoidable reasons of non-participation (like trial refusal) would be equally divided among all participating hospitals.

Planning for recruitment should be an important issue in the preparation period when a trial is designed [7,8]. Attention can also be paid to logistic problems that patients may encounter. To minimize the no-show, reminder notes and telephone calls may remind patients to attend the clinic. A member of the research team, who can provide the information on a low profile with a high level of communication skills and understanding, can support the investigators. This person can deal with practical problems, such as patient's concerns or language barriers. This may contribute to solving the problem of women being less likely to participate in clinical trials [9].

## Conclusion

In conclusion, this study showed that almost three-quarters of our recruitment failures were due to unavoidable reasons. To overcome the remaining avoidable reasons for non-participation and to increase external validity of a trial, it may be contingent upon tracing reasons of clinicians and patients for non-participation as well as by anticipating practical problems that clinicians and patients may encounter during a trial. In the set up of the trial and during the recruitment, communication and information are the key words.

## Competing interests

The author(s) declare that they have no competing interests.

## Authors' contributions

DAMP participated in the design of the study, collected and analyzed the data and wrote the first draft. AJMC performed statistical analyses and took part in the further preparation of the paper. FMH initiated the study, participated in the design of the study, took part in the further preparation of the paper and finalized the manuscript.
